# Crystal structure of (1*R*,5*S*)-*endo*-(8-methyl-8-azoniabi­cyclo­[3.2.1]oct-3-yl)ammonium aqua­tri­chlorido­nitratocopper(II)

**DOI:** 10.1107/S2056989017014633

**Published:** 2017-10-20

**Authors:** Sergey N. Britvin, Andrey M. Rumyantsev

**Affiliations:** a Department of Crystallography, Saint Petersburg State University, Universitetskaya Nab. 7/9, 199034 St Petersburg, Russian Federation; b Department of Genetics and Biotechnology, Saint Petersburg State University, Universitetskaya Nab. 7/9, 199034 St Petersburg, Russian Federation

**Keywords:** crystal structure, tropane, nitro­gen heterocycle, copper(II)complex, isomer separation

## Abstract

The structure of a salt of diprotonated *endo*-3-amino­tropane crystallized with a copper(II) anionic cluster is reported, *viz.* (C_8_H_18_N_2_)[CuCl_3_(NO_3_)(H_2_O)]. Neither ion in the salt has been structurally characterized previously. In the crystal, the ions pack together to form a three-dimensional structure held together by a network of inter­molecular N—H⋯O, O—H⋯Cl and N—H⋯Cl hydrogen-bonding inter­actions. Selective crystallization of the title compound can be considered as a simple method for the separation of the *exo* and *endo* isomers of 3-amino­tropane.

## Abstract 2

The title compound is a salt containing a protonated *endo*-3-amino­tropane cage and a novel anionic copper(II) complex, [CuCl_3_(NO_3_)(H_2_O)]^2−^.

## Chemical context   

**Figure 1:**
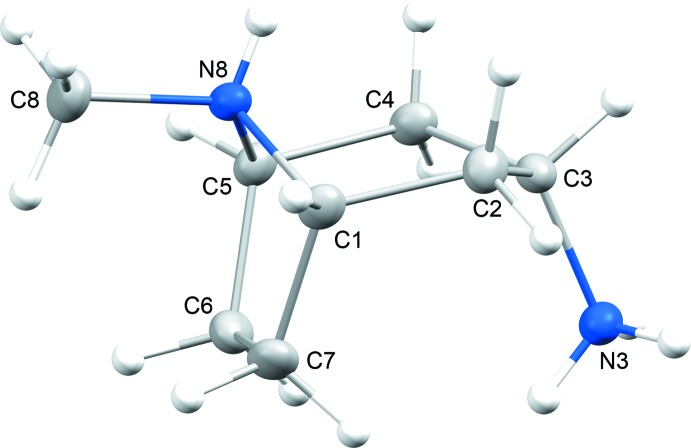
The *endo*-3-amino­tropane skeleton in the crystal structure of **1**. The atomic numbering scheme of the tropane cage is given in accordance with IUPAC nomenclature (Pollini *et al.*, 2006 ▸; Kim *et al.*, 2016 ▸). Displacement ellipsoids are drawn at the 30% probability level. H atoms are shown as fixed-size spheres of 0.15 Å radius.

**Figure 2:**
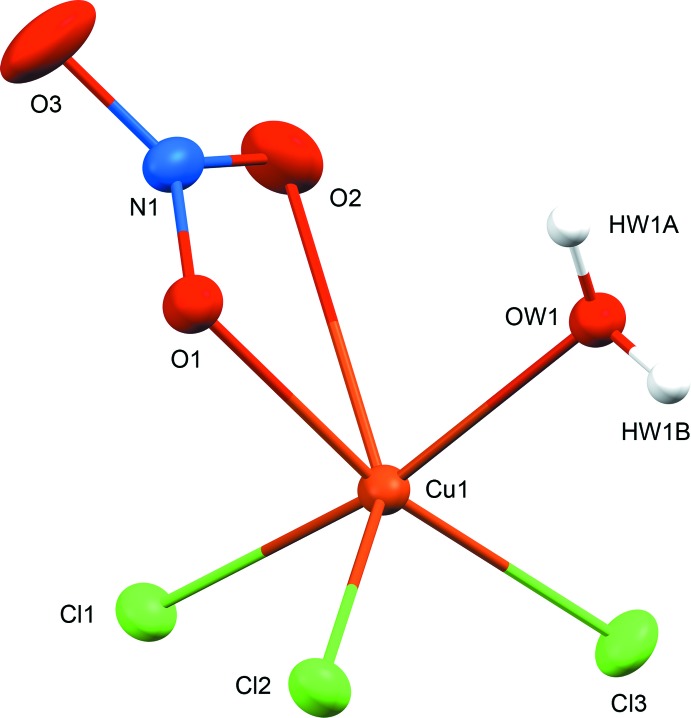
The mol­ecular structure of the novel copper(II) anionic complex, [CuCl_3_(NO_3_)(H_2_O)]^2−^, in **1**. Displacement ellipsoids are drawn at the 30% probability level. H atoms are shown as fixed-size spheres of 0.15 Å radius.

The bicyclic ring of tropane [(1*R*,5*S*)-8-methyl-8-aza­bicyclo­[3.2.1]octa­ne] is the fuctional core of pharmaceutically important alkaloids, such as atropine, hyoscyamine, scopolamine, cocaine and their semisynthetic derivatives (Pollini *et al.*, 2006 ▸; Kim *et al.*, 2016 ▸). As a consequence, there have been a large number of structural studies devoted to tropane-based compounds. It is surprising, however, that some of the simplest derivatives of tropane, such as 3-amino­tropane, have not been structurally characterized in their unsubstituted forms. The structures of other simple and well-known bicyclic organic compounds have been reported only very recently, including 1,4-di­aza­bicyclo­[3.2.1]octane (Britvin *et al.*, 2017 ▸) and 7-aza­bicyclo­[2.2.1]heptane (7-aza­norbornane) (Britvin & Rumyantsev, 2017 ▸). In the course of our ongoing studies of cage-like heterocyclic amines (Britvin & Lotnyk, 2015 ▸; Britvin *et al.*, 2016 ▸), we report herein for the first time the mol­ecular structure of the *endo* isomer of 3-amino­tropane in its proton­ated form (see Scheme). In the title compound, (1*R*,5*S*)-*endo*-(8-methyl-8-azoniabi­cyclo­[3.2.1]oct-3-yl)ammonium aqua­tri­chlorido­nitratocopper(II), **1**, the protonated *endo*-3-amino­tropane skeleton (Fig. 1 ▸) is charge-balanced by the [CuCl_3_(NO_3_)(H_2_O)]^2−^ anion. The anion (Fig. 2 ▸) is the first example of a complex in which a copper(II) centre is coordinated to both nitrate and chloride ligands (as well as water). It is noteworthy that the synthesized compound **1** contains the pure *endo*-3-amino­tropane isomer, whereas the starting material, 3-amino­tropane di­hydro­chloride, comprised a mixture of *exo* and *endo* isomers. Therefore, selective crystallization of **1** reported herein can be recommended as a simple and effective method for the separation of the *exo* and *endo* isomers of 3-amino­tropane.
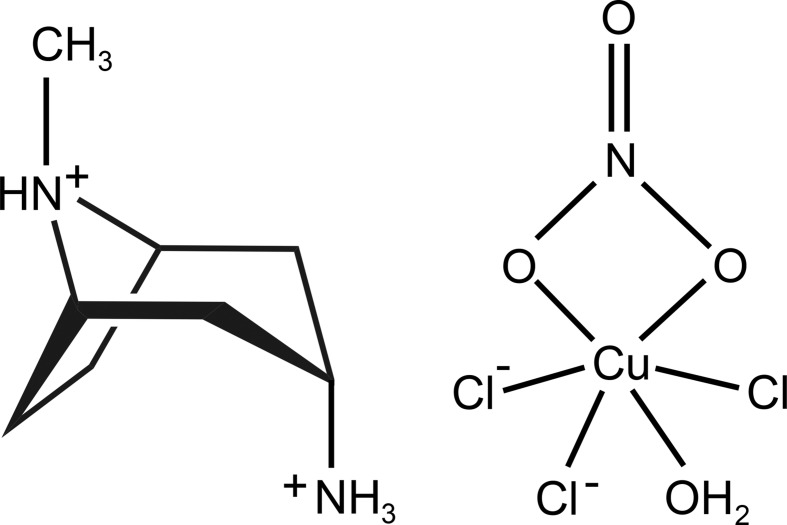


## Structural commentary   

In the structure of **1**, the bicyclic skeleton of 3-amino­tropane has a boat-like conformation with the 3-amino group located in the *endo* position (see Scheme[Chem scheme1] and Fig. 1 ▸). Only five examples of structurally characterized *endo* isomers of 3-amino­tropane have been reported previously (Fludzinski *et al.*, 1987 ▸; Bradley *et al.*, 1992 ▸; Collin *et al.*, 1995 ▸; Omae *et al.*, 2002 ▸), all of which are *N*-3-substituted derivatives. The detailed description of the geometry of the *endo*-3-amino­tropane skeleton in **1** can be found in the supporting information. The 3-amino­tropane unit has two chiral centres located at the C1 (*R*) and C5 (*S*) C atoms. The packing of the 3-amino­tropane mol­ecules in the crystal generates an inversion centre establishing the chiral balance between the alternating 3-amino­tropane units. The anionic moiety, [CuCl_3_(NO_3_)(H_2_O)]^2−^, in the structure of **1** (Fig. 2 ▸) is inter­esting because it is the first reported example of a copper(II) complex coordinated by both chloride and nitrate ligands, in addition to water. The coordination of the Cu^II^ atom by nitrate and water or ammonia ligands is well documented [see, for example, the structures of Cu(NH_3_)_4_(NO_3_)_2_ (Morosin, 1976 ▸; Chukanov *et al.*, 2015 ▸) and Cu(NO_3_)_2_(H_2_O)_2.5_ (Garaj & Gazo, 1969 ▸)]. In addition, a limited number of isolated chloride–aqua and chlorate–aqua complexes of Cu^II^ have been reported as both neutral clusters, *e.g.* [Cu(H_2_O)_2_Cl_2_] (Matkovic *et al.*, 1969 ▸; Bhakay-Tamhane *et al.*, 1980 ▸) and [Cu(H_2_O)_4_(ClO_3_)_2_] (Blackburn *et al.*, 1991 ▸), and anionic complexes, *e.g.* [Cu(H_2_O)_2_Cl_4_]^2−^ (Begley *et al.*, 1988 ▸) and [Cu(H_2_O)_2_Cl_3_]^−^ (Wei & Willett, 1996 ▸). Therefore, the new complex anion, *viz.* [CuCl_3_(NO_3_)(H_2_O)]^2−^, can be considered as a valuable contribution to the aqueous coordination chemistry of copper(II). The geometry of this unusual cluster (Fig. 2 ▸) can be described as a severely distorted octa­hedron, with three Cu—Cl bonds [Cu1—Cl1 = 2.3019 (3), Cu1—Cl2 = 2.5856 (4) and Cu1–Cl3 = 2.2499 (3) Å], one Cu—OH_2_ bond [Cu1—O*W*1 = 2.0646 (10) Å] and two Cu—O bonds from the asymmetrically bonded NO_3_ ligand [Cu1—O1 = 1.9923 (9) Å and the very weak Cu1—O2 = 2.609 (1) Å]. Similar bonding of an NO_3_ group to a Cu^II^ centre, with two distinct bond lengths, has been reported, for example, in Cu(NO_3_)_2_(H_2_O)_2.5_ (Garaj & Gazo, 1969 ▸), anhydrous β-Cu(NO_3_)_2_ (Troyanov *et al.*, 1995 ▸) and (NH_4_)_3_[Cu(NO_3_)_4_](NO_3_) (Morozov *et al.*, 1998 ▸).

## Supra­molecular features   

**Figure 3:**
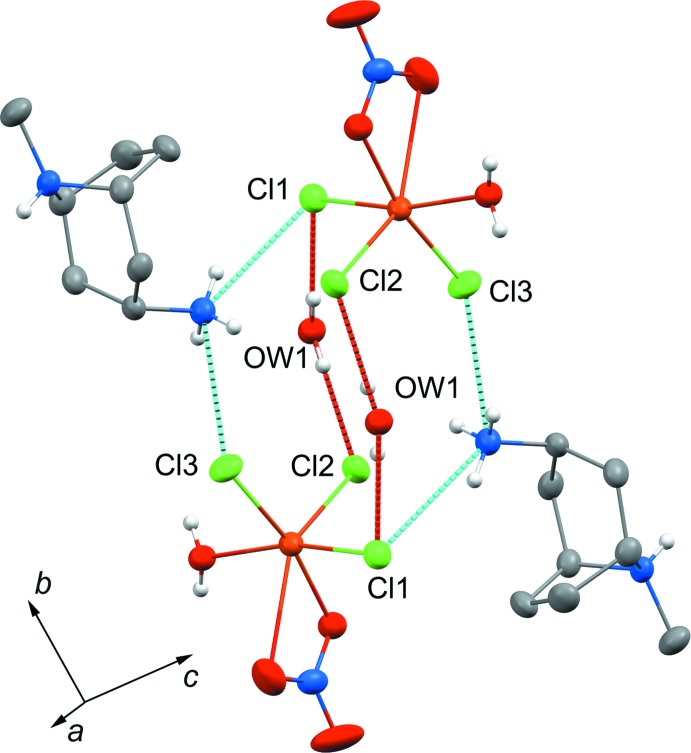
A network of hydrogen bonds maintains the structural integrity of **1**. The bond lengths are given in Table 1 ▸.

The overall integrity of the crystal structure of **1** is achieved *via* a complex three-dimensional network of inter­molecular hydrogen bonds (Fig. 3 ▸). Three types of hydrogen bonding are observed: (i) N—H⋯O inter­actions between the protonated N atom, N8, and the water mol­ecule coordinated to the Cu^II^ atom, (ii) O—H⋯Cl inter­actions involving the same water mol­ecule located between two chloride ions and (iii) N—H⋯Cl inter­actions between the protonated amino group NH_3_^+^ and chloride ions Cl1 and Cl3 (Table 1 ▸).

## Database survey   

Among the 204 structures containing the tropane core in the Cambridge Structural Database (CSD, Version 5.38, latest update May 2017; Groom *et al.*, 2016 ▸), 11 entries contain 3-amino­tropane derivatives, all of which are substituted at the 3-amino group. There are five structures in the CSD and nine in the ICSD (ICSD, 2017 ▸), which contain isolated chloro–aqua complexes of copper(II) (Matkovic *et al.*, 1969 ▸; Bhakay-Tamhane *et al.*, 1980 ▸; Begley *et al.*, 1988 ▸; Wei & Willett, 1996 ▸).

## Synthesis and crystallization   

106.6 mg (0.5 mmol) of 3-amino­tropane di­hydro­chloride (a mixture of the 3-*exo* and 3-*endo* isomers, Sigma–Aldrich) was dissolved in 1 ml of deionized water. 60.4 mg (0.25 mmol) of Cu(NO_3_)_2_·3H_2_O (reagent grade) was dissolved in another 1 ml aliquot of water. On mixing the two solutions, a transparent pale-yellow–green solution was formed. Light-green needles of **1** were grown by slow evaporation of the solution at room temperature.

## Refinement   

H atoms at the protonated N8 and N9 atoms and water mol­ecule O*W*1 were refined freely, whereas H atoms on C atoms were refined based on a riding model. Crystal data, data collection and structure refinement details are summarized in Table 2 ▸.

## Supplementary Material

Crystal structure: contains datablock(s) I. DOI: 10.1107/S2056989017014633/cq2021sup1.cif

Structure factors: contains datablock(s) I. DOI: 10.1107/S2056989017014633/cq2021Isup2.hkl

Supporting information file. DOI: 10.1107/S2056989017014633/cq2021Isup3.mol

CCDC reference: 1571888

Additional supporting information:  crystallographic information; 3D view; checkCIF report

## Figures and Tables

**Table 1:** Hydrogen-bond geometry (Å, °)

*D*—H⋯*A*	*D*—H	H⋯*A*	*D*⋯*A*	*D*—H⋯*A*
N8—H8⋯O*W*1^i^	0.783 (17)	2.236 (17)	2.9600 (15)	154.0 (15)
O*W*1—H*W*1*A*⋯Cl1^ii^	0.79 (2)	2.33 (2)	3.1145 (11)	172.4 (19)
O*W*1—H*W*1*B*⋯Cl2^iii^	0.79 (2)	2.30 (2)	3.0851 (11)	179 (2)

Symmetry codes: (i) 
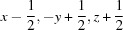
; (ii) 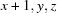; (iii) 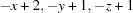.

**Table 2:** Experimental details

Crystal data	
Chemical formula	(C_8_H_18_N_2_)[CuCl_3_(NO_3_)(H_2_O)]
*M*_r_	392.16
Crystal system, space group	Monoclinic, *P*2_1_/*n*
Temperature (K)	150
*a*, *b*, *c* (Å)	6.2464 (3), 13.5674 (6), 17.4584 (8)
β (°)	100.128 (1)
*V* (Å^3^)	1456.50 (12)
*Z*	4
Radiation type	Mo *K*α
μ (mm^−1^)	2.06
Crystal size (mm)	0.25 × 0.20 × 0.15

Data collection	
Diffractometer	Bruker APEXII CCD
Absorption correction	Multi-scan (*SADABS*; Sheldrick, 2015 ▸)
No. of measured, independent and observed [*I* > 2σ(*I*)] reflections	16933, 3523, 3382
*R*_int_	0.012
(sin θ/λ)_max_ (Å^−1^)	0.661

Refinement	
*R*[*F* ^2^ > 2σ(*F* ^2^)], *wR*(*F* ^2^), *S*	0.018, 0.049, 1.05
No. of reflections	3523
No. of parameters	197
H-atom treatment	H atoms treated by a mixture of independent and constrained refinement
Δρ_max_, Δρ_min_ (e Å^−3^)	0.43, −0.31

Computer programs: *APEX2* and *SAINT* (Bruker, 2015 ▸), *SHELXT* (Sheldrick, 2015 ▸), *SHELXL* (Sheldrick, 2015 ▸), *Mercury* (Macrae *et al.*, 2008 ▸), *OLEX2* (Dolomanov *et al.*, 2009 ▸) and *publCIF* (Westrip, 2010 ▸).

## supplementary crystallographic information

### Crystal data

**Table:**

(C_8_H_18_N_2_)[CuCl_3_(NO_3_)(H_2_O)]	*F*(000) = 804
*M**_r_* = 392.16	*D*_x_ = 1.788 Mg m^−^^3^
Monoclinic, *P*2_1_/*n*	Mo *K*α radiation, λ = 0.71073 Å
*a* = 6.2464 (3) Å	Cell parameters from 9865 reflections
*b* = 13.5674 (6) Å	θ = 2.8–35.0°
*c* = 17.4584 (8) Å	µ = 2.06 mm^−^^1^
β = 100.128 (1)°	*T* = 150 K
*V* = 1456.50 (12) Å^3^	Block, green
*Z* = 4	0.25 × 0.20 × 0.15 mm

### Data collection

**Table:**

Bruker APEX-II CCD diffractometer	3523 independent reflections
Radiation source: fine-focus sealed tube	3382 reflections with *I* > 2σ(*I*)
Graphite monochromator	*R*_int_ = 0.012
φ and ω scans	θ_max_ = 28.0°, θ_min_ = 1.9°
Absorption correction: multi-scan SADABS (Sheldrick, 2015)	*h* = −8→8
	*k* = −17→17
16933 measured reflections	*l* = −23→22

### Refinement

**Table:**

Refinement on *F*^2^	0 restraints
Least-squares matrix: full	Hydrogen site location: mixed
*R*[*F*^2^ > 2σ(*F*^2^)] = 0.018	H atoms treated by a mixture of independent and constrained refinement
*wR*(*F*^2^) = 0.049	*w* = 1/[σ^2^(*F*_o_^2^) + (0.0249*P*)^2^ + 0.617*P*] where *P* = (*F*_o_^2^ + 2*F*_c_^2^)/3
*S* = 1.05	(Δ/σ)_max_ = 0.001
3523 reflections	Δρ_max_ = 0.43 e Å^−^^3^
197 parameters	Δρ_min_ = −0.31 e Å^−^^3^

### Special details

**Table:**

Geometry. All esds (except the esd in the dihedral angle between two l.s. planes) are estimated using the full covariance matrix. The cell esds are taken into account individually in the estimation of esds in distances, angles and torsion angles; correlations between esds in cell parameters are only used when they are defined by crystal symmetry. An approximate (isotropic) treatment of cell esds is used for estimating esds involving l.s. planes.

### Fractional atomic coordinates and isotropic or equivalent isotropic displacement parameters (Å^2^)

**Table:**

	*x*	*y*	*z*	*U*_iso_*/*U*_eq_	
C1	0.3006 (2)	0.09472 (10)	0.65317 (7)	0.0272 (3)	
H1	0.1897	0.0452	0.6581	0.033*	
C2	0.2156 (2)	0.19808 (10)	0.66403 (8)	0.0296 (3)	
H2A	0.1662	0.2008	0.7136	0.036*	
H2B	0.0906	0.2101	0.6236	0.036*	
C3	0.3811 (2)	0.28074 (9)	0.66170 (7)	0.0255 (2)	
H3	0.3426	0.3338	0.6949	0.031*	
C4	0.6143 (2)	0.24969 (10)	0.69599 (7)	0.0273 (2)	
H4A	0.7142	0.2906	0.6733	0.033*	
H4B	0.6397	0.2624	0.7515	0.033*	
C5	0.66665 (19)	0.14187 (10)	0.68295 (7)	0.0253 (2)	
H5	0.8146	0.1257	0.7089	0.030*	
C6	0.6311 (2)	0.11169 (11)	0.59713 (8)	0.0323 (3)	
H6A	0.7268	0.0578	0.5892	0.039*	
H6B	0.6579	0.1668	0.5647	0.039*	
C7	0.3908 (3)	0.07923 (11)	0.57794 (8)	0.0347 (3)	
H7A	0.3115	0.1189	0.5361	0.042*	
H7B	0.3798	0.0105	0.5624	0.042*	
N8	0.50100 (18)	0.07935 (8)	0.71434 (6)	0.0239 (2)	
C8	0.5664 (3)	−0.02653 (10)	0.72557 (9)	0.0375 (3)	
H8A	0.4508	−0.0632	0.7418	0.056*	
H8B	0.6950	−0.0313	0.7646	0.056*	
H8C	0.5955	−0.0530	0.6774	0.056*	
N3	0.3612 (2)	0.32304 (9)	0.58084 (7)	0.0285 (2)	
Cu1	0.77174 (2)	0.33409 (2)	0.41227 (2)	0.02287 (5)	
Cl1	0.45571 (5)	0.24722 (2)	0.41328 (2)	0.03028 (7)	
Cl2	0.84844 (5)	0.40007 (2)	0.55338 (2)	0.03060 (7)	
Cl3	0.60443 (6)	0.45734 (2)	0.33880 (2)	0.03453 (8)	
N1	0.94979 (18)	0.15675 (8)	0.40161 (7)	0.0286 (2)	
O1	0.94479 (15)	0.21792 (7)	0.45717 (5)	0.02895 (19)	
O2	0.8758 (2)	0.18232 (10)	0.33464 (7)	0.0465 (3)	
O3	1.0312 (2)	0.07521 (9)	0.41756 (10)	0.0614 (4)	
OW1	1.05693 (16)	0.38867 (8)	0.38441 (6)	0.02756 (19)	
H8	0.484 (3)	0.1001 (12)	0.7547 (10)	0.024 (4)*	
H3A	0.374 (3)	0.2818 (15)	0.5454 (11)	0.040 (5)*	
HW1A	1.164 (3)	0.3573 (15)	0.3938 (11)	0.042 (5)*	
H3B	0.234 (4)	0.3451 (15)	0.5664 (12)	0.050 (6)*	
HW1B	1.080 (3)	0.4426 (17)	0.4001 (12)	0.046 (5)*	
H3C	0.451 (4)	0.3683 (17)	0.5810 (12)	0.052 (6)*	

### Atomic displacement parameters (Å^2^)

**Table:**

	*U*^11^	*U*^22^	*U*^33^	*U*^12^	*U*^13^	*U*^23^
C1	0.0225 (6)	0.0289 (6)	0.0282 (6)	−0.0064 (5)	−0.0009 (5)	0.0005 (5)
C2	0.0210 (6)	0.0344 (7)	0.0346 (7)	0.0010 (5)	0.0081 (5)	0.0051 (5)
C3	0.0302 (6)	0.0244 (6)	0.0232 (5)	0.0014 (5)	0.0080 (5)	0.0003 (4)
C4	0.0276 (6)	0.0269 (6)	0.0261 (6)	−0.0073 (5)	0.0010 (5)	0.0004 (5)
C5	0.0187 (5)	0.0295 (6)	0.0271 (6)	−0.0010 (5)	0.0021 (4)	0.0046 (5)
C6	0.0343 (7)	0.0362 (7)	0.0287 (6)	0.0089 (6)	0.0116 (5)	0.0015 (5)
C7	0.0431 (8)	0.0345 (7)	0.0241 (6)	−0.0017 (6)	−0.0006 (5)	−0.0059 (5)
N8	0.0271 (5)	0.0236 (5)	0.0206 (5)	−0.0025 (4)	0.0031 (4)	0.0005 (4)
C8	0.0471 (8)	0.0242 (6)	0.0394 (7)	0.0014 (6)	0.0029 (6)	0.0052 (5)
N3	0.0303 (6)	0.0289 (6)	0.0267 (6)	0.0020 (5)	0.0059 (5)	0.0035 (4)
Cu1	0.01998 (8)	0.02360 (8)	0.02488 (8)	0.00238 (5)	0.00351 (6)	0.00206 (5)
Cl1	0.02233 (14)	0.03309 (16)	0.03568 (16)	−0.00169 (11)	0.00582 (11)	0.00026 (12)
Cl2	0.03173 (16)	0.03230 (16)	0.02900 (15)	−0.00508 (12)	0.00873 (12)	−0.00633 (12)
Cl3	0.03283 (16)	0.02662 (15)	0.04061 (18)	0.00540 (12)	−0.00329 (13)	0.00498 (13)
N1	0.0203 (5)	0.0242 (5)	0.0404 (6)	−0.0028 (4)	0.0033 (4)	−0.0055 (4)
O1	0.0292 (5)	0.0287 (5)	0.0280 (4)	0.0026 (4)	0.0024 (4)	0.0006 (4)
O2	0.0488 (7)	0.0601 (8)	0.0312 (5)	−0.0034 (6)	0.0087 (5)	−0.0084 (5)
O3	0.0518 (7)	0.0272 (6)	0.0952 (11)	0.0092 (5)	−0.0145 (7)	−0.0147 (6)
OW1	0.0238 (5)	0.0249 (5)	0.0342 (5)	0.0009 (4)	0.0057 (4)	0.0006 (4)

### Geometric parameters (Å, º)

**Table:**

C1—H1	0.9800	C7—H7B	0.9700
C1—C2	1.5231 (19)	N8—C8	1.4970 (17)
C1—C7	1.5318 (19)	N8—H8	0.783 (17)
C1—N8	1.5103 (16)	C8—H8A	0.9600
C2—H2A	0.9700	C8—H8B	0.9600
C2—H2B	0.9700	C8—H8C	0.9600
C2—C3	1.5305 (18)	N3—H3A	0.85 (2)
C3—H3	0.9800	N3—H3B	0.85 (2)
C3—C4	1.5333 (18)	N3—H3C	0.83 (2)
C3—N3	1.5086 (16)	Cu1—Cl1	2.3019 (3)
C4—H4A	0.9700	Cu1—Cl2	2.5856 (4)
C4—H4B	0.9700	Cu1—Cl3	2.2499 (3)
C4—C5	1.5247 (18)	Cu1—O1	1.9923 (9)
C5—H5	0.9800	Cu1—OW1	2.0646 (10)
C5—C6	1.5313 (18)	N1—O1	1.2811 (15)
C5—N8	1.5132 (16)	N1—O2	1.2292 (17)
C6—H6A	0.9700	N1—O3	1.2289 (16)
C6—H6B	0.9700	OW1—HW1A	0.79 (2)
C6—C7	1.543 (2)	OW1—HW1B	0.79 (2)
C7—H7A	0.9700		
			
C2—C1—H1	110.7	C6—C7—H7A	110.7
C2—C1—C7	114.95 (11)	C6—C7—H7B	110.7
C7—C1—H1	110.7	H7A—C7—H7B	108.8
N8—C1—H1	110.7	C1—N8—C5	101.59 (9)
N8—C1—C2	107.63 (10)	C1—N8—H8	110.9 (12)
N8—C1—C7	101.74 (10)	C5—N8—H8	109.7 (12)
C1—C2—H2A	108.6	C8—N8—C1	113.48 (10)
C1—C2—H2B	108.6	C8—N8—C5	113.39 (11)
C1—C2—C3	114.80 (10)	C8—N8—H8	107.7 (12)
H2A—C2—H2B	107.5	N8—C8—H8A	109.5
C3—C2—H2A	108.6	N8—C8—H8B	109.5
C3—C2—H2B	108.6	N8—C8—H8C	109.5
C2—C3—H3	106.6	H8A—C8—H8B	109.5
C2—C3—C4	112.83 (10)	H8A—C8—H8C	109.5
C4—C3—H3	106.6	H8B—C8—H8C	109.5
N3—C3—C2	111.03 (11)	C3—N3—H3A	115.4 (13)
N3—C3—H3	106.6	C3—N3—H3B	109.3 (15)
N3—C3—C4	112.70 (10)	C3—N3—H3C	109.5 (15)
C3—C4—H4A	108.6	H3A—N3—H3B	102.9 (18)
C3—C4—H4B	108.6	H3A—N3—H3C	109.8 (19)
H4A—C4—H4B	107.5	H3B—N3—H3C	110 (2)
C5—C4—C3	114.79 (10)	Cl1—Cu1—Cl2	100.684 (12)
C5—C4—H4A	108.6	Cl3—Cu1—Cl1	94.122 (14)
C5—C4—H4B	108.6	Cl3—Cu1—Cl2	105.990 (13)
C4—C5—H5	110.8	O1—Cu1—Cl1	89.92 (3)
C4—C5—C6	113.86 (11)	O1—Cu1—Cl2	84.38 (3)
C6—C5—H5	110.8	O1—Cu1—Cl3	167.91 (3)
N8—C5—C4	107.79 (10)	O1—Cu1—OW1	86.88 (4)
N8—C5—H5	110.8	OW1—Cu1—Cl1	164.17 (3)
N8—C5—C6	102.33 (10)	OW1—Cu1—Cl2	94.43 (3)
C5—C6—H6A	110.8	OW1—Cu1—Cl3	86.14 (3)
C5—C6—H6B	110.8	O2—N1—O1	118.83 (11)
C5—C6—C7	104.90 (11)	O3—N1—O1	118.42 (13)
H6A—C6—H6B	108.8	O3—N1—O2	122.75 (13)
C7—C6—H6A	110.8	N1—O1—Cu1	107.35 (8)
C7—C6—H6B	110.8	Cu1—OW1—HW1A	119.7 (15)
C1—C7—C6	105.36 (10)	Cu1—OW1—HW1B	111.5 (15)
C1—C7—H7A	110.7	HW1A—OW1—HW1B	109 (2)
C1—C7—H7B	110.7		
			
C1—C2—C3—C4	−33.81 (15)	C6—C5—N8—C1	−46.59 (12)
C1—C2—C3—N3	93.81 (13)	C6—C5—N8—C8	75.54 (13)
C2—C1—C7—C6	86.42 (13)	C7—C1—C2—C3	−56.77 (15)
C2—C1—N8—C5	−74.06 (12)	C7—C1—N8—C5	47.14 (12)
C2—C1—N8—C8	163.87 (11)	C7—C1—N8—C8	−74.93 (13)
C2—C3—C4—C5	33.45 (15)	N8—C1—C2—C3	55.76 (14)
C3—C4—C5—C6	57.78 (14)	N8—C1—C7—C6	−29.54 (13)
C3—C4—C5—N8	−55.00 (13)	N8—C5—C6—C7	27.56 (13)
C4—C5—C6—C7	−88.46 (13)	N3—C3—C4—C5	−93.28 (13)
C4—C5—N8—C1	73.74 (12)	O2—N1—O1—Cu1	7.68 (14)
C4—C5—N8—C8	−164.13 (11)	O3—N1—O1—Cu1	−173.23 (11)
C5—C6—C7—C1	1.22 (14)		

### Hydrogen-bond geometry (Å, º)

**Table:**

*D*—H···*A*	*D*—H	H···*A*	*D*···*A*	*D*—H···*A*
N8—H8···O*W*1^i^	0.783 (17)	2.236 (17)	2.9600 (15)	154.0 (15)
O*W*1—H*W*1*A*···Cl1^ii^	0.79 (2)	2.33 (2)	3.1145 (11)	172.4 (19)
O*W*1—H*W*1*B*···Cl2^iii^	0.79 (2)	2.30 (2)	3.0851 (11)	179 (2)

Symmetry codes: (i) *x*−1/2, −*y*+1/2, *z*+1/2; (ii) *x*+1, *y*, *z*; (iii) −*x*+2, −*y*+1, −*z*+1.
